# Application of Plasma Metabolomic Biomarker Panels in Early Diagnosis and Disease Staging of Alzheimer’s Disease

**DOI:** 10.3390/metabo16060377

**Published:** 2026-05-30

**Authors:** Jiao Chen, Xuhui Chen, Ting Chen, Jun Hu

**Affiliations:** 1 Department of Neurology, Peking University Shenzhen Hospital, Shenzhen 518036, China; chenjiao@pkuszh.com.cn (J.C.); xuhuichen@pkuszh.com (X.C.); 2 Department of Neurology, Peking University First Hospital, Beijing 100034, China; 3 Department of Neurology, Shenzhen Second People’s Hospital, Shenzhen 518036, China; 4 Shenzhen Key Laboratory for Neuronal Structural Biology, Shenzhen Peking University—The Hong Kong University of Science and Technology Medical Center, Shenzhen 518036, China

**Keywords:** Alzheimer’s disease (AD), metabonomics, plasma, metabolic biomarkers, cognitive function, Chinese population

## Abstract

**Highlights:**

**What are the main findings?**

- A panel of 16 plasma metabolites effectively distinguished early-stage Alzheimer’s disease and mild cognitive impairment from healthy controls and other cognitive disorders in a cohort of 172 participants.
- Specific metabolite levels exhibited progressive changes associated with memory decline severity across different stages of the disease.

**What are the implications of the main findings?**

- Blood-based metabolite testing offers a simpler, more accessible alternative to invasive or expensive current diagnostic methods for early AD detection and monitoring.
- These findings suggest that metabolic profiling could facilitate earlier intervention and improved outcomes for patients and families affected by AD.

**Abstract:**

**Background**: Previous metabolomics studies on Alzheimer’s disease (AD) have predominantly focused on Western populations, leaving Chinese cohorts and disease stage-specific data largely unexplored. **Objectives**: To characterize metabolic alterations across different clinical stages of AD in a Chinese population and identify early diagnostic biomarkers. **Methods**: We enrolled 172 participants, including patients with AD, mild cognitive impairment (MCI), subjective cognitive decline (SCD), vascular cognitive impairment (VCI), and healthy controls (HC). Untargeted metabolomics (LC-MS and GC-MS) was performed on plasma samples, integrated with Mini-Mental State Examination (MMSE) and Montreal Cognitive Assessment (MoCA) assessments. Data were analyzed using multivariate statistics, pathway enrichment, and ROC modeling. **Results:** Distinct metabolic profiles emerged across disease stages, with phospholipids, ceramides, and glucose metabolites prominently enriched in glycerophospholipid, sphingolipid, and glucose pathways. A 16-metabolite panel achieved robust discrimination between AD+MCI and HC+VCI+SCD (AUC = 0.804). Specific metabolites, including ceramides, dihydroceramides, phosphatidylinositol, phosphatidylcholine, and glycodeoxycholic acid, correlated significantly with cognitive function and disease progression. **Conclusions**: This study reveals stage-specific metabolic dysregulation in Chinese AD patients and identifies potential plasma biomarkers for early detection, offering insights into AD pathogenesis. Trial registration number ChiCTR2400092653.

## 1. Introduction

Alzheimer’s disease (AD) is a complex and deteriorating neurodegenerative disease, which is now considered to be the main cause of dementia worldwide, bringing a great burden to the medical system and families [1]. In 2020, more than 55 million people globally were affected by AD and related dementia, with projections indicating 78 million cases by 2030, primarily attributable to population aging [2]. The pathological features of AD have been clearly defined, which are mainly extracellular deposits formed by misfolded β-amyloid (Aβ) peptide and intracellular neurofibrillary tangles (NFTs) caused by abnormal hyperphosphorylation and aggregation of tau protein [3,4]. These two diseases work together, slowly leading to synaptic damage, decreased synaptic connections, and a wide range of neuronal degeneration, finally making the cognitive function of patients gradually deteriorate [5].

Although substantial progress has been made in elucidating AD pathogenesis, its diagnosis and treatment remain challenging. Effective early detection methods are limited, and AD symptoms substantially overlap with those of healthy controls (HC), subjective cognitive decline (SCD), vascular cognitive impairment (VCI), and mild cognitive impairment (MCI), complicating differential diagnosis [6,7]. Currently, diagnostic methods such as cerebrospinal fluid Aβ and tau detection and amyloid positron emission tomography (amyloid-PET) exhibit certain specificity, but their widespread clinical application and screening are limited by invasiveness, high costs, and equipment accessibility [8]. Therefore, the development of non-invasive biomarkers with high sensitivity and specificity is crucial not only for early AD detection but also for distinguishing among HC, SCD, VCI, MCI, and AD, thereby optimizing diagnostic and therapeutic strategies [9,10].

Metabolomics, a rapidly evolving field within high-throughput omics, enables comprehensive profiling of small-molecule metabolites (molecular weight: 50–1500 Da) in tissues and biofluids. This approach systematically reveals alterations in metabolic networks associated with disease states [11]. As of April 2023, the Human Metabolome Database (HMDB, URL: https://hmdb.ca) has included more than 220,000 metabolite entries covering both water-soluble and lipid-soluble metabolites, providing comprehensive basic data support for research in this field [12]. Detected substances include naturally occurring metabolites and externally introduced substances, which show various physical and chemical characteristics and stability in biological systems. As direct substrates and products of biochemical reactions, their concentration fluctuations reflect actual physiological states [13]. Compared with the characteristics that gene expression is regulated by epigenetics and protein activity depends on post-translational modifications, metabolites, as direct products of biochemical processes, are more likely to establish associations with specific phenotypes, providing key molecular clues for elucidating the pathophysiological mechanisms of diseases [14]. By capturing the characteristics of small-molecule metabolites in a panoramic manner, metabolomics can present a comprehensive picture of biochemical activity and cellular physiological status in organisms, and it has now become a core technical means for clarifying the metabolic basis of AD. Numerous studies have confirmed that AD patients generally have key metabolic abnormalities, such as lipid metabolism imbalance, mitochondrial energy production disorders, amino acid homeostasis disruption, and redox balance disturbance [15,16,17].

However, current evidence is predominantly derived from Western cohorts, with insufficient metabolomic studies focusing on the Chinese population and different disease stages of AD. This limitation hinders a comprehensive understanding of the pathophysiological mechanisms underlying AD. To address these gaps, we implemented a metabolomic strategy combined with the Mini-Mental State Examination (MMSE) and Montreal Cognitive Assessment (MoCA) [18] scales in a cohort of 172 participants. By performing correlation analyses between untargeted metabolomics based on liquid chromatography–mass spectrometry (LC-MS) and clinical assessments, we aimed to construct an early diagnostic model for AD and identify metabolites associated with AD severity.

## 2. Materials and Methods

### 2.1. Study Participants and Grouping

This case–control study was conducted at the Department of Neurology at Peking University Shenzhen Hospital. The target sample size was determined based on prior metabolomics biomarker studies and established statistical practice, aiming for a minimum of 30 participants per group in the primary comparative analysis to support robust multivariate statistical modeling. Of 183 initially recruited participants, 11 were excluded prior to metabolomic profiling due to insufficient plasma volume or failure to meet quality control criteria (Supplementary Figure S1).

A total of 172 participants were enrolled, including 60 clinically diagnosed AD patients and 33 HCs; the remaining participants consisted of 47 cases with VCI and other cognitive abnormalities (21 with VCI, 26 with other conditions), 9 patients with MCI, and 23 subjects with SCD (see Supplementary Figure S1 for the study flow chart).

All procedures were performed in accordance with the Declaration of Helsinki. The study protocol was approved by the Institutional Review Board (IRB) (approval number: 2024-151, 30 October 2024), and written informed consent was obtained from all participants prior to enrollment.

Participants were recruited based on predefined inclusion and exclusion criteria:

Inclusion criteria for AD group: Aged 45–90 years; clinically diagnosed with AD based on the AT(N) framework, confirmed by positive blood or cerebrospinal fluid biomarkers or PET-CT results.

Inclusion criteria for HCs: Cognitively normal, confirmed by screening with the MMSE.

AD severity classification: Stratified by MMSE scores or Clinical Dementia Rating (CDR) scores: MMSE scores: 0–9 = severe AD, 10–20 = moderate AD, 21–24 = mild AD, 25–26 = MCI, ≥27 = SCD; CDR scores: 0 = healthy, 0.5 = questionable dementia, 1 = mild dementia, 2 = moderate dementia, 3 = severe dementia.

Exclusion criteria: Comorbid major psychiatric disorders (e.g., schizophrenia, bipolar disorder); comorbid neurological diseases other than AD (e.g., Parkinson’s disease, stroke); comorbid vascular diseases or active malignancies; chronic renal/hepatic/pulmonary/cardiac dysfunction; antibiotic use within 3 months before enrollment; major gastrointestinal diseases or surgery within 1 year before enrollment; or refusal to provide informed consent.

The characteristic data of participants, including gender and onset age, were obtained through standardized case report forms to fully understand their clinical situation. Clinical diagnosis was established prior to metabolomic analysis.

### 2.2. Sample Collection and Storage

We took 5 mL of whole blood samples from volunteers and put them into a vacuum EDTA blood collection tube made by BD Diagnostics (Franklin Lakes, NJ, USA). These samples were immediately put into a refrigerated centrifuge at 4 °C and centrifuged at 1500× *g* for 15 min. After centrifugation, the upper plasma was aliquoted into 1.5 mL Eppendorf tubes and stored at −80 °C for subsequent analysis.

Plasma samples were collected concurrently with clinical and neuropsychological assessments to ensure that metabolomic profiles reflected the same disease state as the reference standard diagnosis. Participants were instructed to fast overnight (≥12 h) before blood collection to minimize acute dietary effects on plasma metabolite levels. No adverse events related to blood sampling for metabolomic analysis or clinical diagnostic procedures were reported during this study. Venipuncture was performed by trained phlebotomists using standard aseptic technique. Neuropsychological assessments and diagnostic imaging were conducted according to routine clinical protocols.

### 2.3. Metabolite Extraction and Quality Control

In order to prevent sequence errors, we disrupted the sequence and randomly arranged samples from different experimental groups for testing. Sample processing was performed by technicians blinded to group allocation. In each project, we mixed all samples to make quality control (QC) samples, and these QC samples and research samples could go through exactly the same pretreatment together.

Metabolites were extracted according to the Plasma Extraction Standard Operating Procedure [19]. Only samples for GC-MS analysis were subjected to derivatization prior to testing: derivatization of standard substances yielded various derivatized products, and the derivatization efficiency was evaluated by determining the derivatized products of representative metabolites in QC standards [20]. No indeterminate results were encountered for the index test or reference standard.

### 2.4. Instrumental Analysis

To obtain comprehensive plasma metabolic profiles, two types of instruments were used for data acquisition in this study, which were functionally subdivided into three analytical platforms for the detection of metabolic features in plasma samples:

GC-MS platform: Thermo Fisher TRACE 1610 gas chromatograph (Waltham, MA, USA) coupled with Thermo Fisher ISQ 7610 mass spectrometer (Waltham, MA, USA). Ultra performance liquid chromatography–mass spectrometry (UPLC-MS) platform: Waters ACQUITY UPLC I-Class liquid chromatograph (Milford, MA, USA) coupled with Thermo Fisher Q Exactive mass spectrometer (Waltham, MA, USA), which was further subdivided into the high-resolution LC-MS Polar platform and LC-MS Lipid platform.

### 2.5. Data Preprocessing and Annotation

High-quality annotated peaks for subsequent analysis were required to have a detection rate > 50% in samples, and these underwent preprocessing, including cross-platform deduplication and derivatization adduct filtering. The data set was processed as follows: First, ion peaks with missing values in more than half of the samples were removed. Then, missing values in each compound were replaced with one-fifth of the smallest positive value of that compound. Then, the data were standardized using the sum normalization method. Finally, to improve data distribution, square root transformation was applied to the standardized values.

Identification of compounds was initially performed using the UlibMS metabolite reference database (Metanotitia Inc., Harbin, China) based on *m*/*z*, retention time, and MS/MS spectral matching. Putative identifications were subsequently cross-validated against public databases, including the Human Metabolome Database (HMDB, version 5.0) and LIPID MAPS for structural confirmation. To ensure the stability and reliability of the results, only the most abundant adduct form in each group of compounds was selected, regardless of other adduct forms. The selection was based on two criteria: the average RT of all samples in the whole group and the average mass-to-charge ratio (*m*/*z*), which were used to match each important peak.

Processing and annotation of GC-MS data: Established protocols for biological sample analysis, the in-house MT UlibMS metabolite reference database, and targeted analysis methods were employed. Both identification and quantification were performed using gas chromatography–quadrupole mass spectrometry data. This instrument was equipped with a high-sensitivity ion source, and analysis focused on retention time, characteristic ion *m*/*z*, and intensity value.

### 2.6. Statistical Analysis

Multivariate statistical analysis was performed to compare the metabolic characteristics of different groups. Orthogonal Partial Least Squares Discriminant Analysis (OPLS-DA) was employed for classification, and variable importance in projection (VIP) scores were calculated from the model. Metabolites with VIP scores greater than 1 were selected, as they were considered important for distinguishing different groups and could help identify potential biomarkers.

For two-group comparisons, Student’s *t*-test was used; for multi-group comparisons, analysis of variance (ANOVA) was used. Metabolites meeting the criteria of |log_2_(fold change, FC)| ≥ 0.38 and *p* < 0.05 were considered significantly different. Volcano plots were generated using the ggplot2 package (version 3.5.1) in R (version 4.3.3) to visualize these differential metabolites. Heatmaps were constructed using the pheatmap package in R, and relative abundance patterns of all metabolites and expression differences between different groups were displayed through hierarchical clustering.

To identify enriched metabolic pathways, the clusterProfiler package (version 4.10.0) in R was used, evaluating enrichment based on log_10_ (*p* value) and enrichment ratio.

Multivariate receiver operating characteristic (ROC) analysis was performed to evaluate the classification performance of metabolite combinations. Models were developed using repeated random sampling to identify optimal biomarker combinations. Performance was assessed by the area under the ROC curve (AUC), with values approaching 1 indicating more accurate classification.

Pearson correlation coefficients were calculated to assess correlations between metabolite concentrations and clinical indicators, particularly MMSE and MoCA scores, as well as AD disease stage.

## 3. Results

### 3.1. Demographic and Disease Characteristics

Demographic and disease characteristics of HC, AD, MCI, SCD, and VCI groups are summarized in Table 1. The mean ages ranged from 62.2 ± 9.4 years (MCI) to 69.3 ± 12.5 years (VCI), with no statistically significant differences among the five groups (*p* > 0.05). Regarding gender distribution, the male/female ratios were 20/13 in HC, 30/30 in AD, 7/2 in MCI, 12/11 in SCD, and 23/24 in VCI. For disease staging in the AD group, there were 18 cases of mild AD, 26 cases of moderate AD, and 16 cases of severe AD; disease staging was not applicable to the other groups. The flow of participant recruitment, screening, and group allocation is presented in Supplementary Figure S1. Collectively, these results indicate that the five groups were well-matched in terms of core demographic indicators (age and gender), establishing a robust foundation for subsequent metabolomic difference analysis and inter-group comparisons.

### 3.2. Differential Metabolite Screening and Diagnostic Model Construction for AD vs. HCs

**Figure 1:**
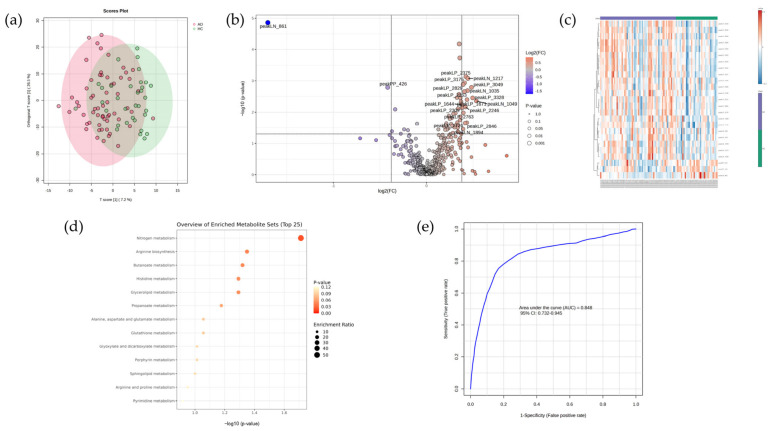
Metabolomic differential analysis and diagnostic model construction between healthy controls (HC) and Alzheimer’s dementia (AD) patients. (**a**) OPLS-DA score plot shows distinct separation of metabolic profiles between HC (green) and AD (red) groups, indicating significant metabolic differences between the two groups; (**b**) volcano plot displays differentially expressed metabolites between HC and AD groups, with log_2_FC on the x-axis and −log_10_ (*p* value) on the y-axis; red dots represent significantly upregulated metabolites, and blue dots represent significantly downregulated metabolites. (**c**) Heatmap of differentially expressed metabolites, where rows represent metabolites and columns represent samples. The color changes from blue to red, representing the expression level of metabolites from low to high, so it can be clearly seen that the metabolic characteristics of HC group and AD group are different. (**d**) This bubble chart shows the top 25 metabolic pathways rich in differential metabolites. The abscissa is the enrichment ratio, the ordinate is the name of the pathway, the color of the bubble corresponds to the *p* value, and the size of the bubble represents the number of different metabolites in each pathway. (**e**) This ROC curve shows the AD diagnostic model constructed with key differential metabolites.

The OPLS-DA analysis (Figure 1a) shows that the metabolic profiles of the HC and AD groups exhibit partial overlap but clear separation, indicating substantial metabolic alterations between groups. Based on the criteria of VIP > 1.5 and *p* < 0.05, 42 significantly altered metabolites were identified (Figure 1b). Among these, 17 were upregulated, and two were downregulated in AD samples. The upregulated metabolites were predominantly lipids, including ceramides (e.g., Hex1Cer d42:1, Hex1Cer d41:1, Hex1Cer d42:1O, Hex1Cer d42:2, Hex1Cer d40:1, CerG3GNAc1 d44:1), phosphatidylinositols (PI 36:2, PI 38:5, PI 36:3, PI 32:1), phosphatidylcholines (PC 32:0e, PC 40:4e, PC 36:1e, PC 34:2e), lysophosphatidylcholines (LysoPC 18:0e, LysoPC 20:0), and sphingomyelin (SM t34:1). The downregulated metabolites were glucose and D-2-aminoadipic acid. The clustered heatmap (Figure 1c) revealed distinct expression patterns of these differential metabolites between the two groups.

Pathway enrichment analysis (Figure 1d) based on differential metabolites demonstrated significant enrichment in nitrogen metabolism, arginine biosynthesis, and butanoate metabolism (*p* < 0.05), with additional enrichment trends observed in histidine metabolism; glycerolipid metabolism; propanoate metabolism; alanine, aspartate and glutamate metabolism; glutathione metabolism; glyoxylate and dicarboxylate metabolism; porphyrin metabolism; sphingolipid metabolism; arginine and proline metabolism; and pyrimidine metabolism. Collectively, these findings suggest that metabolic dysregulation may contribute to AD pathophysiology: nitrogen metabolism and arginine biosynthesis play key roles in nitric oxide production and neurotransmitter homeostasis, and their dysregulation may contribute to neuroinflammation and synaptic dysfunction in AD, whereas butanoate metabolism produces short-chain fatty acids that modulate neuroinflammation and blood–brain barrier integrity, suggesting impaired microbial–gut–brain axis communication in AD patients.

A panel of five metabolites (Glucose, D-2-Aminoadipic acid, Hex1Cer d42:1 (d18:1/24:0), PI 38:4, PI 40:4) was selected via LASSO regression. The multivariate ROC model (Figure 1e) constructed with this panel achieved an AUC of 0.848, indicating effective discrimination between HC and AD samples.

### 3.3. Differential Metabolite Screening and Diagnostic Model Construction for AD and MCI vs. HCs

**Figure 2:**
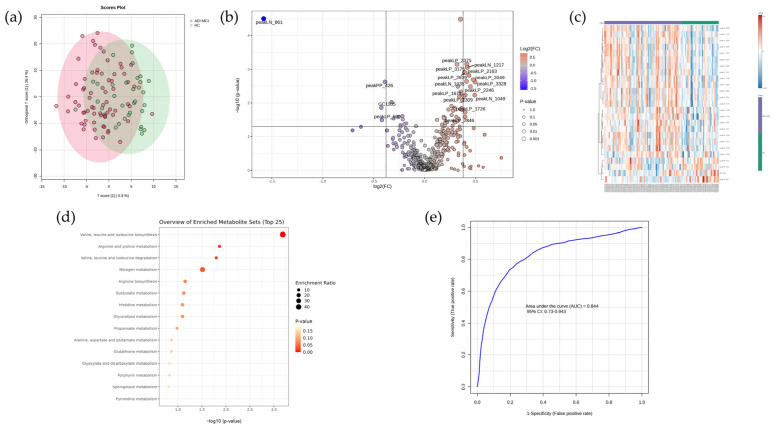
Metabolomic differential analysis and diagnostic model construction between HC and combined AD and MCI patients. (**a**) OPLS-DA score plot shows distinct separation of metabolic profiles between HC (green) and AD+MCI (red) groups, indicating significant metabolic differences between the two groups. (**b**) Volcano plot displays differentially expressed metabolites between HC and AD+MCI groups, with log_2_FC on the x-axis and −log_10_ (*p* value) on the y-axis. Red dots represent significantly upregulated metabolites, and blue dots represent significantly downregulated metabolites. (**c**) Heatmap of differentially expressed metabolites, where rows represent metabolites and columns represent samples. The color gradient from blue to red indicates metabolite expression levels from low to high, clearly distinguishing the metabolic characteristics of HC and AD+MCI groups. (**d**) Bubble plot of the top 25 enriched metabolic pathways for differential metabolites. The x-axis shows the enrichment ratio, and the y-axis lists the names of pathways; the color of the dot represents *p* value, and the size of the dot represents how many differential metabolites there are in each pathway. (**e**) This diagram shows the ROC curve of the AD+MCI diagnostic model derived from the key differential metabolites.

Figure 2a of OPLS-DA shows that the metabolic characteristics of the HC group and the AD+MCI group are obviously different. Based on the judging criteria VIP > 1.5 and *p* < 0.05, we identified 42 different metabolites (Figure 2b). Among them, 14 metabolites have obviously increased in AD+MCI samples, and four metabolites have decreased. Up-regulated metabolites were predominantly lipids, including multiple ceramides (Hex1Cer d42:1, Hex1Cer d41:1, Hex1Cer d42:1O, Hex1Cer d42:2, Hex1Cer d40:1, CerG3GNAc1 d44:1), phosphatidylinositols (PI 36:2, PI 38:5, PI 36:3), phosphatidylcholines (PC 40:4e, PC 34:2e), lysophosphatidylcholines (LysoPC 18:0e, LysoPC 20:0), and sphingomyelin (SM t34:1).

Metabolites with reduced content include Glucose, D-2-Aminoadipic acid, 2-Hydroxybutanoic acid and TAG 54:3. Figure 2c shows the expression levels of the top 25 metabolites selected through statistical analysis in different groups: lipid substances in the blue region, such as ceramides and phospholipids, have low expression in healthy control samples. However, the expression of amino acid derivatives, organic acids and monosaccharides in the green area decreased in AD and mild cognitive impairment patients, showing obvious group characteristics.

We analyzed metabolites with great changes and found that several important metabolic pathways had obvious changes (Figure 2d). These pathways include: the synthesis of valine, leucine and isoleucine; metabolism of arginine and proline; decomposition of valine, leucine and isoleucine; and nitrogen metabolism. In addition, we also found that some pathways are likely to change, such as arginine synthesis, butanoate metabolism, histidine metabolism, glycerolipid metabolism, propanoate metabolism, alanine, aspartate and glutamate metabolism, glutathione metabolism, glyoxylate and dicarboxylate metabolism, porphyrin metabolism, sphingolipid metabolism, and pyrimidine metabolism. Generally speaking, these results show that the fundamental cause of AD and MCI is the disorder of metabolic function in the body. Among them, the three most seriously affected pathways are very important for the development of the disease: the synthesis and decomposition of valine, leucine and isoleucine, which are very important for maintaining the balance of branched-chain amino acids (BCAAs) in the body. Problems with these pathways may hinder the connection between nerve cells and the production of neurotransmitters in the brains of AD and MCI patients, which may be one of the reasons why cognitive ability begins to deteriorate at an early stage. Arginine and proline metabolism and Nitrogen metabolism regulate nitric oxide production and ammonia detoxification, and abnormalities in these pathways may exacerbate neuroinflammation and oxidative stress. The enrichment of additional pathways further implicates disrupted microbial–gut–brain axis communication, antioxidant defense, neurotransmitter balance, and membrane integrity in the transition from MCI to AD.

A panel of five metabolites (2-Hydroxyvaleric acid, Glucose, D-2-Aminoadipic acid, Hex1Cer d42:1 (d18:1/24:0), and PI 38:4) was selected via LASSO regression. The multivariate ROC model (Figure 2e) constructed with this panel achieved an AUC of 0.844, which indicated effective discrimination between HC and AD+MCI samples.

### 3.4. Differential Metabolite Screening and Diagnostic Model Construction for AD+MCI vs. HC+VCI+SCD

**Figure 3:**
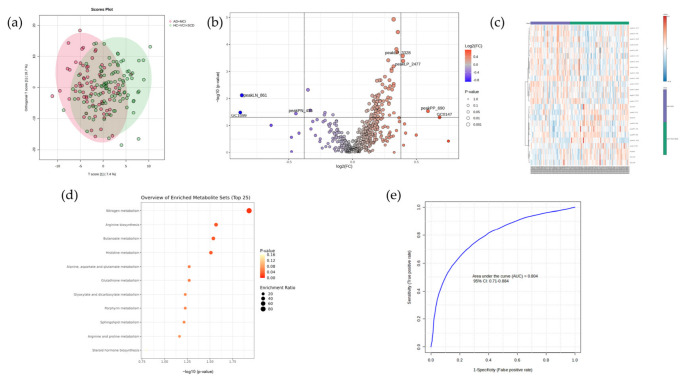
Metabolomic differential analysis and diagnostic model construction between AD+MCI and HC+VCI+SCD groups. (**a**) OPLS-DA score plot shows distinct separation of metabolic profiles between AD+MCI (red) and HC+VCI+SCD (green) groups, indicating significant metabolic differences between the two groups. (**b**) Volcano plot displays differentially expressed metabolites between AD+MCI and HC+VCI+SCD groups, with log_2_FC on the x-axis and −log_10_ (*p* value) on the y-axis. Red dots represent significantly upregulated metabolites, and blue dots represent significantly downregulated metabolites. (**c**) Heatmap of differentially expressed metabolites, where rows represent metabolites and columns represent samples. The color gradient from blue to red indicates metabolite expression levels from low to high, clearly distinguishing the metabolic characteristics of AD+MCI and HC+VCI+SCD groups. (**d**) Bubble plot of the top 25 enriched metabolic pathways for differential metabolites. The x-axis shows the enrichment ratio, and the y-axis lists the name of the pathway; the color of the dot represents *p* value, and the size of the dot represents how many differential metabolites there are in each pathway. (**e**) This diagram shows the ROC curve of the AD+MCI diagnostic model derived from the key differential metabolites.

The OPLS-DA score chart (Figure 3a) shows that the metabolic characteristics of the AD+MCI group and HC+VCI+SCD group are partially mixed, but they are obviously different, which indicates that there are systematic differences in metabolism between the two groups. According to the standards of VIP > 1.5 and *p* < 0.05, Figure 3b shows 23 metabolites with significant differences. The results showed that four metabolites in AD+MCI samples obviously increased, and three metabolites decreased.

The increased metabolites include ceramide (Hex1Cer D42: 1O (d18: 1/24: 0O), CER T41:0 (T16:0/25:0)), 6-aminoxanoic acid and Sucrose; the decreased metabolites are Glucose, Benzoic acid and Apocynin. Figure 3c shows the expression of 25 metabolites with the greatest difference: the lipids in the blue cluster (such as ceramide and phosphatidylinositol) are lowly expressed in HC+VCI+SCD samples, while the amino acid derivatives, organic acids and monosaccharides in the green cluster are lowly expressed in AD+MCI samples, showing an obvious group-specific trend.

We performed pathway enrichment analysis on 22 metabolites with obvious changes (Figure 3d) and found that the pathways of Nitrogen metabolism, Arginine biosynthesis, butyric acid metabolism and Histidine metabolism have changed greatly (*p* < 0.05). Additionally, Alanine, Aspartate and glutamate metabolism, Glutathione metabolism, glyoxylate and dicarboxylate metabolism, Porphyrin metabolism, Sphingolipid metabolism, Arginine and proline metabolism and Steroid hormone biosynthesis have also changed significantly. Generally speaking, these results indicate that the pathogenesis of AD+MCI is related to extensive metabolic imbalance, and the four most affected pathways are particularly critical to the development of the disease. Specifically, nitric oxide and Arginine biosynthesis control the production of nitric oxide and the balance of neurotransmitters, and their imbalance can aggravate the neuroinflammation and synaptic dysfunction in the brain of AD+MCI. Butanoate metabolism mediates the synthesis of short-chain fatty acids, a key regulator of gut–brain axis cross-talk; dysregulation of this pathway leads to impaired signaling transduction among the gut microbiota, intestine and brain, a perturbation linked to exacerbated neuroinflammation and Aβ accumulation. Likewise, dysregulation of histidine metabolism impairs the host’s capacity to regulate neurotransmission and combat oxidative stress in the central nervous system. If sphingolipid is not processed properly, it can make ceramides pile up and lead to nerve cell death; also, if the processing of glutathione is unbalanced, it means that our antioxidant system is weakened, so the oxidative damage can be more serious. The enrichment of additional pathways, such as Alanine, aspartate and glutamate metabolism; Arginine and proline metabolism; and Steroid hormone biosynthesis further implicates disrupted neurotransmitter homeostasis, impaired neuroprotective signaling, and weakened steroid-mediated neuroprotection in AD+MCI pathogenesis.

A panel of 16 metabolic biomarkers (Ribose, L-Asparagine, Benzoic acid, 2-Hydroxyvaleric acid, Glucose, Ascorbate, 6-Aminohexanoic acid, Nicotinamide, Cer t41:0 (t16:0/25:0), TAG 57:3, Hex1Cer d41:1 (d18:1/23:0), TAG 55:5, PI 38:4, PC 40:6, PE 38:5, PG 34:1) was selected to construct a multivariate ROC model (Figure 3e). This model achieved an AUC of 0.804, thus indicating effective discriminatory ability between the AD+MCI and HC+VCI+SCD groups. The diagnostic performance of this model, including sensitivity, specificity, positive predictive value (PPV), and negative predictive value (NPV), is summarized in Table 2.

### 3.5. Differential Metabolite Screening and Diagnostic Model Construction for MCI vs. HCs

**Figure 4:**
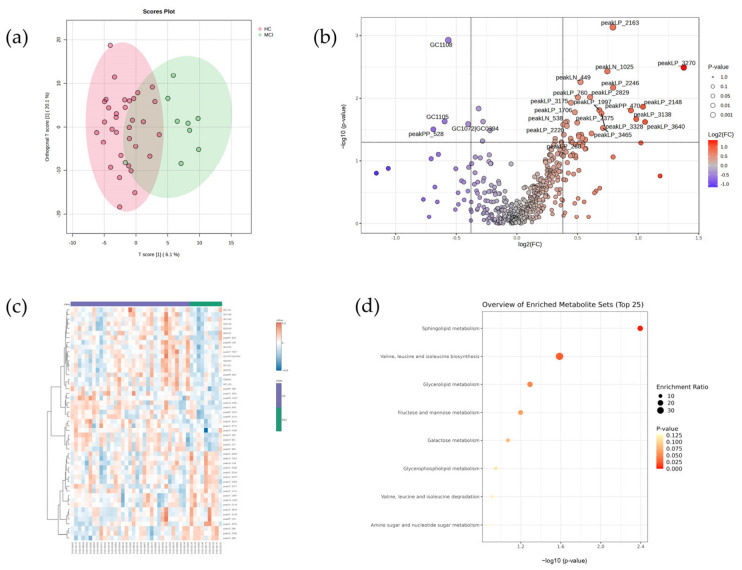
Metabolomic differential analysis and diagnostic model construction between HC and MCI groups. (**a**) OPLS-DA score plot shows distinct separation of metabolic profiles between HC (green) and MCI (red) groups, indicating significant metabolic differences between the two groups. (**b**) Volcano plot displays differentially expressed metabolites between HC and MCI groups, with log_2_FC on the x-axis and −log_10_ (*p* value) on the y-axis. Red dots represent significantly upregulated metabolites, and blue dots represent significantly downregulated metabolites. (**c**) Heatmap of differentially expressed metabolites, where rows represent metabolites and columns represent samples. The color changes from blue to red, representing the expression level of metabolites from low to high, so it can be clearly seen that the metabolic characteristics of HC group and MCI group are very different. (**d**) Bubble plot of the enriched metabolic pathways for differential metabolites. The horizontal axis is the enrichment ratio, the vertical axis is the name of the pathway, the color of the dot represents the *p* value, and the size of the dot corresponds to the number of differential metabolites found in each pathway.

The OPLS-DA score chart (Figure 4a) shows that although the metabolic characteristics of the HC group and the MCI group are somewhat mixed, there is a clear directional separation, which indicates that there are persistent metabolic differences between the two groups. Figure 4b initially detected that the expression of 24 metabolites changed, among which 20 metabolites increased and four decreased in MCI samples. After that, according to the standards of VIP > 1.5 and *p* < 0.05, 12 important metabolites were selected for further study.

Upregulated metabolites were predominantly lipid species, including multiple ceramides (Cer d43:3 (d19:1/24:2), Hex1Cer d42:2 (d18:2/24:0)|Hex1Cer d42:2 (d18:1/24:1), Cer t42:1 (t18:1/24:0)|Cer t42:1 (t17:0/25:1), Hex1Cer d41:1 (d18:1/23:0), Cer d42:3 (d18:2/24:1), Hex2Cer d34:2 (d18:2/16:0), Hex1Cer d42:1 (d18:1/24:0), Hex1Cer d42:1O (d18:1/24:0O)), sphingomyelins (SM t34:1 (t18:0/16:1), SM(d18:1/17:0)), phosphatidylinositol (PI 38:4), lysophosphatidylcholine (LysoPC 18:0e), phosphatidylcholine (PC 34:0e), acylcarnitines (AcCa 14:2, AcCa 10:0, AcCa 10:1, AcCa 12:0, L-Octanoylcarnitine), and triacylglycerols (TAG 54:7, TAG 54:5). Downregulated metabolites included 2-Hydroxyvaleric acid, 2-Hydroxybutanoic acid, 4-Fluoro-L-threonine|N-Methylnicotinic acid, and Galactose 1|Mannose 1. The clustered heatmap (Figure 4c) shows the expression of 50 metabolites with the most obvious changes. We found that lipid components such as ceramides and sphingomyelins are less in HC samples. However, in MCI samples, the contents of some small organic acids, amino acid derivatives and carbohydrates have decreased, which revealed distinct group-specific expression profiles across the two cohorts.

Figure 4d displays their enriched metabolic pathways. The results showed that they were closely related to Sphingolipid metabolism and the synthesis of valine, leucine, and isoleucine (*p* < 0.05). In addition, we also found some obvious enrichment trends, mainly in the metabolism of Glycerolipid, fructose and mannose, galactose, and glycerophospholipid; the degradation of valine, leucine and isoleucine; and the metabolism of amino sugar and nucleotide sugar. Together, these results show that MCI is accompanied by a wide range of metabolic abnormalities, of which two pathways with the most obvious changes are crucial to the initial stage of cognitive decline. The disorder of Sphingolipid metabolism is manifested by an increase in ceramide concentration, which can promote the accumulation of Aβ and abnormal phosphorylation of tau protein, which in turn can damage synaptic activity and neuron health. Similarly, the biosynthesis of branched-chain amino acids, including valine, leucine and isoleucine, has changed, destroying their own metabolic balance, which is very important for producing neurotransmitters and maintaining synaptic plasticity, so it is likely to play a role in early cognitive defects. The additional changes in the metabolism of Glycerolipid and Glycerophospholipid point to the deterioration of neuronal membrane flexibility and the disruption of cell signal transmission. In addition, the irregular metabolism of Fructose, mannose and Galactose means that there are problems in energy production and neuroprotective mechanisms, and all these factors work together to promote the process from MCI to AD.

### 3.6. Metabolite Expression Patterns and Linear Correlation in AD Stages

**Figure 5:**
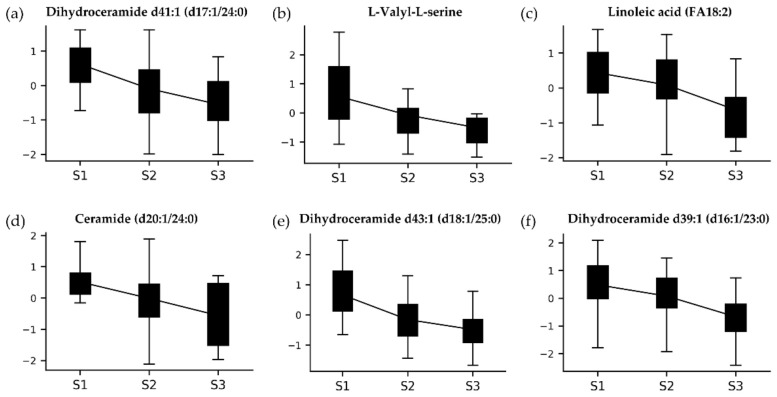
The expression patterns and linear relationships of six different metabolites in different stages of AD. The data shows the levels of these metabolites in patients with mild (S1), moderate (S2), and severe (S3) AD. (**a**) Dihydroceramide d41: 1 (d17: 1/24: 0); (**b**) L-Valyl-L-serine; (**c**) linoleic acid (FA18:2); (**d**) ceramide (d20: 1/24: 0); (**e**) dihydroceramide d43: 1 (d18: 1/25: 0) and (**f**) dihydroceramide d39: 1 (d16: 1/23: 0).

We performed a linear correlation analysis on metabolomic data from patients with AD, who were stratified into three stages: mild (S1), moderate (S2), and severe (S3). The results showed that six metabolites were significantly correlated with disease progression (Figure 5), among which dihydroceramides were the main components. Specifically, the mean relative abundance of Dihydroceramide d41:1 (d17:1/24:0) decreased from 0.582 (max: 1.571, min: −0.725) at S1 to −0.518 (max: 0.842, min: −2.000) at S3; L-Valyl-L-serine decreased from 0.491 (max: 2.712, min: −1.082) at S1 to −0.455 (max: −0.015, min: −1.527) at S3; Linoleic acid (FA18:2) decreased from 0.438 (max: 1.645, min: −1.064) at S1 to −0.573 (max: 0.835, min: −1.812) at S3; Ceramide (d20:1/24:0) decreased from 0.457 (max: 1.785, min: −0.165) at S1 to −0.531 (max: 0.705, min: −2.000) at S3; Dihydroceramide d43:1 (d18:1/25:0) decreased from 0.552 (max: 2.455, min: −0.655) at S1 to −0.468 (max: 0.785, min: −1.685) at S3; and Dihydroceramide d39:1 (d16:1/23:0) decreased from 0.463 (max: 2.105, min: −1.785) at S1 to −0.615 (max: 0.735, min: −2.435) at S3. These metabolites exhibited significant negative correlations with AD progression, suggesting potential roles in disease pathogenesis: decreased dihydroceramide levels may reflect compromised neuronal membrane integrity, thereby exacerbating synaptic dysfunction and neuronal death; linoleic acid is an essential fatty acid, and its reduction may impair membrane fluidity and antioxidant capacity; and decreased L-valyl-L-serine may interfere with neurotransmitter synthesis and neuroprotective mechanisms. These alterations may contribute to progressive AD deterioration from mild to severe stages.

### 3.7. Correlation Analysis Between Metabolites and Cognitive Function Scales (MMSE, MoCA)

**Figure 6:**
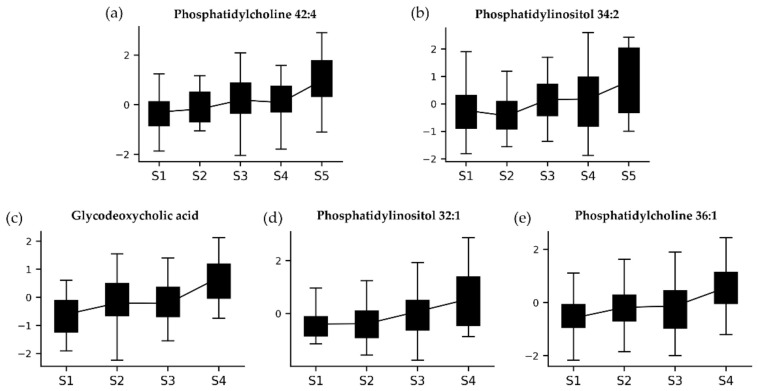
Correlation analysis between metabolites and cognitive function scales (MMSE, MoCA) with linear correlation analysis. The figure shows the correlation between the expression levels of 5 metabolites and cognitive scores: (**a**) Phosphatidylcholine 42:4 (MMSE); (**b**) Phosphatidylinositol 34:2 (MMSE); (**c**) Glycodeoxycholic acid (MoCA); (**d**) Phosphatidylinositol 32:1 (MoCA); (**e**) Phosphatidylcholine 36:1 (MoCA).

Linear correlation analysis of metabolomic data with MMSE scores, using a cutoff of |R| > 0.28 (Figure 6a,b), revealed that the mean expression level of Phosphatidylcholine 42:4 increased across cognitive stages S1 to S5, from −0.282 (max: 1.241, min: −1.848) at S1 to 0.945 (max: 2.862, min: −1.055) at S5. Similarly, the mean expression level of Phosphatidylinositol 34:2 also showed an increasing trend from −0.248 (max: 1.895, min: −1.792) at S1 to 0.816 (max: 2.443, min: −0.992) at S5, both exhibiting significant positive linear correlations with MMSE scores. These findings point to a potential contribution of phospholipid metabolic dysfunction to cognitive decline in AD, likely via impairments to neuronal membrane integrity and synaptic plasticity. Phosphatidylcholine, an essential structural constituent of neuronal membranes, elicits a reduction in membrane fluidity when its expression is downregulated, thereby impairing the efficiency of synaptic transmission; phosphatidylinositol, a key player in cellular signal transduction cascades, can disrupt the activation of neuroprotective signaling pathways when metabolically dysregulated, which in turn exacerbates neuronal damage and subsequent cognitive impairment.

We correlated metabonomic data with MoCA scores using linear correlation analysis (|R| > 0.3, Figure 6c–e) and identified three metabolic markers. The results showed that the average expression level of Glycodeoxycholic acid increased from −0.525 (range: 0.612 to −1.895) at the S1 stage to 0.584 (range: 2.148 to −0.742) at the S4 stage. Similarly, the ratio of Phosphatidylinositol 32: 1 increased from −0.985 (range: 0.334 to −1.625) at S1 to 0.324 (range: 2.412 to −1.405) at S4, while the ratio of Phosphatidycholine 36: 1 increased from −0.578 (range: 1.124 to −2.155) at S1 to 0.495 (range: 2.438 to −1.185) at S4. Glycodeoxycholic acid is a metabolite of intestinal flora. These metabolic changes suggest impaired gut–brain axis signaling, which may worsen neuroinflammation and Aβ accumulation, thus accelerating AD pathology. The altered phospholipids also point to a link between phospholipid metabolism disorder and early cognitive impairment in AD, making them potential early indicators of cognitive decline.

## 4. Discussion

In order to fill an important gap in the metabolomics research of AD, most of the previous studies focused on the Western population, while the data of Chinese and different stages of the disease were insufficient. Our study recruited 172 participants, including MCI, SCD, VCI and HCs. We combined the non-targeted metabolomics analysis of LC-MS and GC-MS, and the clinical evaluation using MMSE and MoCA scales, and then carried out strict data preprocessing, multivariate statistical methods, pathway enrichment analysis and ROC modeling. Our study found that different stages of disease have unique metabolic characteristics, such as key metabolites such as phospholipids, ceramides and glucose-related compounds. At the same time, it was found that these metabolites were significantly enriched in glycerophospholipid metabolism, sphingolipid metabolism and glucose metabolism. We also developed an effective early diagnosis model, which has strong discrimination ability, and used 16 metabolic biomarkers (AUC = 0.804) to distinguish AD+MCI from HC+VCI+SCD. We found that there is a group of specific metabolites, mainly ceramides, dihydroceramides, phosphatidylinositol, phosphatidylcholine and glycodeoxycholic acid, which are obviously related to cognitive ability and disease severity. Together, these results reveal the metabolic abnormalities of Chinese AD patients at different stages and put forward some possible metabolic biomarkers, which can help to identify the disease early and better understand its pathogenesis.

Dysregulated lipid metabolism modulates the onset and progression of AD via multiple pathways [21]. Firstly, dysregulated lipid metabolism impairs the metabolism of Aβ and tau proteins: abnormal lipid metabolism disrupts Aβ production, aggregation and clearance, leading to neuritic plaque formation and neuronal damage; it also alters the activities of tau-associated kinases and phosphatases, causing excessive tau hyperphosphorylation, neurofibrillary tangle formation and further neuronal structural and functional impairment [22]. Secondly, it triggers neuroinflammation and oxidative stress: lipid peroxides from abnormal lipid metabolism activate inflammatory signaling pathways, elevating inflammatory cytokine release and inducing neuroinflammation that damages neurons, disrupts the blood–brain barrier and exacerbates AD pathogenesis [23]. Additionally, it enhances oxidative stress, causing excessive free radical production and impaired antioxidant defense, which induces neuronal lipid, protein and DNA damage, accelerating neuronal death and AD progression [24].

Under AD pathological conditions, alterations in certain lipids, such as PI, in the central nervous system (CNS) exhibit similar trends to those observed in peripheral tissues [25,26]. The relationship between peripheral lipid changes and CNS processes in AD can be interpreted through several mechanisms. First, peripheral lipids may reflect CNS-derived lipids released into the bloodstream following neuronal damage and blood–brain barrier (BBB) disruption. In AD, BBB integrity is progressively compromised, allowing CNS-derived metabolites to leak into peripheral circulation [23]. Second, systemic lipid dysregulation may directly affect brain lipid homeostasis through circulating lipoproteins and lipid-transfer proteins that cross the BBB. Third, the brain and periphery share common regulatory mechanisms for lipid metabolism, including insulin signaling, inflammation, and oxidative stress pathways that operate across both compartments [27,28,29]. The regulatory dissociation effect of the brain–peripheral axis on central and peripheral lipid metabolism in AD may explain some of the apparent discrepancies between our plasma findings and reported brain tissue alterations. For instance, while PC species are generally downregulated in brain tissue and RBC membranes in AD [30,31,32], we observed that PC 42:4 and PC 36:1 in plasma were positively correlated with cognitive scores. These differences likely reflect the distinct metabolic pressures operating in the CNS versus peripheral compartments, as well as compensatory systemic responses to central lipid depletion.

The differential lipids identified in this study possess unique acyl chain structures, and their changes extend far beyond simple quantitative alterations. The enrichment of saturated or monounsaturated sphingoid bases within long-chain fatty acid ceramides (C23–C25) indicates enhanced membrane rigidity and increased lipid raft stability, which facilitates Aβ oligomer formation and receptor aggregation [33]. The reduction of highly polyunsaturated phospholipids (PC 40:6, PE 38:5) reflects insufficient de novo synthesis or dietary supplementation of DHA/EPA, consequently diminishing cell membrane fluidity and anti-inflammatory signaling capacity [34,35]. Collectively, these structural characteristics support the notion that AD involves not merely global elevation or reduction of lipid levels but rather selective remodeling of acyl chain length, saturation status, and headgroup composition. Such remodeling plays a significant role in altering membrane biophysical properties, signal transduction, and neuroinflammation.

Beyond their structural roles in membrane bilayers, phospholipid alterations profoundly affect lipid raft organization and APP processing. Lipid rafts are cholesterol- and sphingolipid-enriched microdomains that serve as platforms for the assembly of signaling molecules and the proteolytic processing of membrane proteins [36]. In our study, the enrichment of long-chain ceramides with saturated sphingoid bases (C23–C25) likely contributes to the stabilization of lipid rafts, creating a more rigid membrane environment that promotes the clustering of β-secretase (BACE1) and facilitates amyloidogenic APP processing [33]. In synaptic membranes, sphingolipids modulate neurotransmitter receptor activity and regulate the trafficking of NMDA and AMPA receptors [37]. The reduction in highly polyunsaturated phospholipids (PC 40:6, PE 38:5) observed in our study compromises membrane fluidity at synaptic terminals, thereby impairing vesicle fusion, neurotransmitter release, and postsynaptic receptor mobility. These changes negatively affect synaptic transmission and APP processing, ultimately leading to increased Aβ generation [38]. Furthermore, glycerophospholipids constitute a core class of signaling lipids that play essential roles in membrane structure and intracellular signal transduction [39]. PC is a key structural component of cell membranes, and disruption of its lateral homeostasis reduces the efficiency of calcium-dependent vesicle fusion, thereby compromising membrane fluidity and elasticity and ultimately impairing membrane function [40].

The consistent alterations observed in phosphatidylcholines, phosphatidylinositols, and lysophospholipids in our study likely reflect fundamental disturbances in phospholipid remodeling pathways, most notably the Lands’ cycle. This cycle involves the deacylation and reacylation of glycerophospholipids through the sequential actions of phospholipase A2 (PLA2) enzymes and lysophospholipid acyltransferases (LPLATs), serving as the primary mechanism by which the acyl chain composition of membrane phospholipids is maintained [40]. In AD, dysregulation of the Lands’ cycle leads to the accumulation of lysophospholipids—the transient intermediates of this remodeling process—and alterations in the final acyl chain composition of PC and PI species. Lysophospholipids are transient metabolites generated during glycerophospholipid remodeling [40]. For instance, lysophospholipids can be produced through phospholipase A2 activation [41] and increase with elevated oxidative stress [42]. A recent DESI-mass spectrometry imaging study demonstrated that lysophospholipids co-aggregate with Aβ deposits in the brain tissue of AD patients [43]. This finding suggests a potential direct interaction between lysophospholipids and Aβ; the elevated LysoPC levels observed in the plasma of AD patients in this study may provide insight into this phenomenon. Lysophospholipids and other glycerophospholipid metabolites exert pro-inflammatory effects by activating astrocytes and microglia to stimulate cytokine release, thereby further exacerbating oxidative stress and neuroinflammation. The underlying mechanisms include upregulation of cytosolic phospholipase A2 (cPLA2) isoforms, cyclooxygenase (COX), and nitric oxide synthase (NOS) [44,45]. In the present study, glycerophospholipid metabolism-related metabolites were closely associated with AD disease discrimination and progression severity, which is consistent with previous reports associating membrane phospholipid dysregulation with AD pathogenesis [46]. The AD-related glycerophospholipids identified in this study were primarily PC, lysophospholipids, and PI, suggesting that disruption of the Lands’ cycle may be a central feature of AD-associated phospholipidopathy.

Notably, we observed that the plasma concentration of phosphatidylinositol 38:4 (PI 38:4) was significantly upregulated in the disease groups in the comparisons of HC vs. AD, HC vs. AD+MCI, and HC+VCI+SCD vs. AD+MCI. It exhibited a characteristic expression pattern in AD converters and patients with MCI and was incorporated as a core biomarker into the three ROC plasma diagnostic models we constructed, serving as a key metabolite for distinguishing AD and AD+MCI from various control populations. PI and its phosphorylated derivatives serve specialized functions in intracellular signal transduction, most notably the role of PIP3 in activating the AKT pathway [47]. The PI3K/AKT signaling pathway plays a critical role in CNS development, survival, and metabolic regulation, particularly in neuronal growth, differentiation, and apoptosis. Activation of this pathway promotes glucose uptake through GLUT4 translocation, inhibits apoptosis via BAD phosphorylation, and suppresses GSK-3β activity, thereby reducing tau hyperphosphorylation. However, this pathway is frequently suppressed in AD patients [48], leading to impaired neuronal survival signaling, enhanced tau pathology, and reduced insulin sensitivity. As one of the abundant phosphatidylinositol (PI) subtypes in plasma [49], PI 38:4 is an essential member of glycerophospholipids and plays an irreplaceable role in neuronal membrane structural composition and cellular signal transduction. The upregulation of PI 38:4 in AD plasma may reflect a compensatory response to PI3K/AKT pathway suppression, wherein cells increase PI availability to sustain downstream signaling. Alternatively, elevated PI 38:4 may indicate dysregulated phosphoinositide turnover, as the conversion of PI to PI(4,5)P2 and subsequently to PIP3 is impaired in AD, leading to substrate accumulation.

In a study of coronary atherosclerosis using a familial hypercholesterolemia swine model, Slijkhuis et al. identified PI 38:4 as a core component driving atherosclerotic lipid signatures in negative ionization mode; it colocalizes with inflammatory cells in atherosclerotic plaques and acts as a critical lipid involved in the development of coronary atherosclerotic plaques [50]. Multiple studies have demonstrated a significant association between hyperlipidemia and the risk of AD onset. In a 3-year follow-up of elderly residents in Taiyuan, Cox regression analysis identified hyperlipidemia as a risk factor for progression from MCI to AD (RR = 2.22, 95% CI: 1.29–3.82) [51]. Another study analyzed serum samples from 45 AD patients and 44 healthy controls and found that the serum total cholesterol (TC) levels in AD patients were significantly higher than those in the healthy control group (*p* < 0.05), suggesting that dyslipidemia may be implicated in the development of AD [52]. These results show that PI 38:4 (a common lipid molecule in blood) is not only a key factor in the formation of coronary plaque but also serves as a strong diagnostic marker. It links hyperlipidemia to AD risk through lipid metabolism pathways. Two subtypes of phosphatidylinositol, PI 34:2 and PI 32:1, are correlated with the MMSE and MoCA scores, respectively. PI 34:2 has been demonstrated to be downregulated in the disease group in rat studies focusing on vascular dementia [53], whereas PI 32:1 was found to exhibit dysregulated expression in patients with chronic obstructive pulmonary disease (COPD) [54]. However, direct evidence supporting the specific associations of these two subtypes with AD remains scarce to date, which warrants further in-depth investigation.

Although PC 42:4 and PC 36:1 have some common characteristics with PI, they have one obvious difference: these phospholipids are positively correlated with MMSE and MoCA scores. However, when comparing healthy people with early patients, there was no statistically significant difference in their expression changes, so they were not selected as plasma biomarkers for early detection models. Existing studies have confirmed that PC exhibits a marked downregulation pattern in AD [30], with the most abundant PC species in the brain undergoing aberrant turnover accompanied by accelerated catabolism [31]. A study enrolling 146 AD patients, 45 Parkinson’s disease (PD) patients, 30 coronary artery disease (CAD) patients and 39 healthy elderly individuals (control group) demonstrated that PC levels in red blood cell (RBC) membranes of AD patients are concomitantly reduced with plasmalogens, which represents a core pathological feature of phospholipid metabolism in AD [32]. This finding is inconsistent with our observation that PC 42:4 and PC 36:1 are positively correlated with cognitive scores in AD patients, which may be attributed to the differences in the focused PC subtypes, detected biological samples (brain/RBC membranes vs. plasma) and disease stages (moderate-severe vs. early-mild) across studies, as well as the regulatory dissociation effect of the brain–peripheral axis on central and peripheral PC metabolism in AD. The underlying mechanisms driving the aforementioned subtype specificity and spatiotemporal metabolic heterogeneity of PC in AD remain to be further investigated in depth.

Lipids are major components of cell membranes and play crucial roles in regulating physiological processes such as membrane synthesis, energy supply, and cell signaling [55]. In synaptic membranes, sphingolipids modulate neurotransmitter receptor activity [37]. In lipid rafts, sphingolipids, together with cholesterol, regulate the activity of transmembrane proteins, including APP and BACE1 [36]. Sphingolipids can also function as lipid second messengers, regulating cellular stress resistance, proliferation, differentiation, and survival in the nervous system [56]. Previous studies have found that sphingomyelin levels are significantly decreased in the plasma of AD patients, whereas region-specific changes are observed in the brain, with increases in gray matter, cerebrospinal fluid, and cerebellum [26,57], and decreases in frontolimbic regions [58]. In this study, elevated plasma sphingomyelin levels were observed in AD patients. Such discrepancies in sphingomyelin levels may be influenced by the heterogeneous activity of lipid metabolic enzymes across different compartments [59]. Furthermore, extensive evidence indicates that major lipid alterations affect APP processing. Sphingolipids, which are abundant in lipid rafts and myelin sheaths, play important roles in APP processing. Increased membrane diacylglycerol content embedded in lipid rafts promotes APP β-cleavage mediated by the low-affinity p75 neurotrophin receptor (p75NTR), thereby increasing Aβ generation [60].

Ceramide, as a bioactive sphingolipid, has been consistently found to be elevated in both central and peripheral sites in AD patients [61,62], which is consistent with the results of this study. The alterations in ceramides and dihydroceramides across disease stages observed in our study can be understood through two major metabolic pathways: the sphingomyelin turnover pathway and the de novo synthesis pathway. In the turnover pathway, ceramide and sphingomyelin are metabolically interrelated and can be hydrolyzed and converted into each other; therefore, enhanced sphingomyelinase (SMase) activity mediating the conversion of sphingomyelin to ceramide is considered a partial explanation for elevated ceramide levels in AD patients [63]. In the de novo synthesis pathway, ceramide is generated from serine and palmitoyl-CoA via the action of serine palmitoyltransferase (SPT) and subsequent enzymes in the endoplasmic reticulum. Upregulation of de novo ceramide synthesis has been documented in AD brain tissue and is associated with ER stress and apoptosis. Beyond the sphingomyelin pathway, ceramide can also be generated through alternative salvage pathways involving the reacylation of sphingosine [64]. Consequently, the causal relationships among the interconversion of these molecules under AD pathological conditions remain incompletely understood. Ceramide has also been demonstrated to promote Aβ formation by modulating APP cleavage and enhancing BACE1 activity [65]. At the cellular level, ceramide accumulation disrupts mitochondrial function, increases reactive oxygen species production, and promotes neuronal apoptosis through caspase activation. Sphingomyelin and ceramide are important components of extracellular vesicles such as exosomes [66,67], and studies have shown that cell-derived exosomes can accelerate Aβ formation and propagation [68], suggesting that ceramide-enriched exosomes may serve as vehicles for spreading pathological Aβ species between neurons.

We further found that glucose was consistently downregulated in the disease groups across the comparisons of HC vs. AD, HC vs. AD+MCI, and HC+VCI+SCD vs. AD+MCI and served as a key component in all three diagnostic models. The observed reduction in circulating glucose levels carries significant biological implications for AD pathophysiology. Cerebral glucose hypometabolism is a prominent pathological abnormality in the preclinical stage of AD, detectable decades before clinical symptom onset. As the key pathway for glucose catabolism, glycolysis may play a central role in AD pathogenesis. Glycolysis is essential for a variety of neuronal activities in the brain, and reduced glycolytic flux is correlated with the severity of amyloid-β and tau pathology [69]. The accumulation of glucose in the brain despite peripheral hypoglycemia suggests that disruption of the glycolysis process—rather than glucose availability—may be the primary driver of cerebral energy failure in AD.

Extensive studies have demonstrated that insulin resistance plays an important role in the onset and progression of AD [27,28,29]. Insulin resistance enhances neuroinflammation [70], triggering amyloid-β accumulation through increased reactive oxygen species production [71,72]. Oxidative stress associated with insulin resistance also dysregulates glycogen synthase kinase 3-β (GSK-3β), leading to increased tau phosphorylation [73]. This evidence suggests that diabetic pathway dysfunction may be a fundamental feature of AD at its initial manifestation. Clinical studies have revealed that both fasting plasma glucose and glycated hemoglobin (HbA1c) levels are significantly lower in patients with AD dementia than in non-dementia individuals [74], and only 8.8% of patients with diabetes mellitus develop AD dementia, in contrast with 27.9% of non-diabetic individuals [75]. The paradox of peripheral glucose reduction coexisting with cerebral glucose accumulation highlights the centrality of insulin resistance and impaired glucose utilization in AD pathogenesis.

Our study has several strengths, including the high robustness of the model attributed to the inclusion of multiple metabolites, a relatively large sample size, and its origin from a single Chinese cohort; we evaluated a comprehensive panel of lipid subtypes, and to minimize the use of invasive procedures in clinical practice, we validated our findings using paired plasma samples. Furthermore, we included patients with VCI and SCD as controls, which enhanced the specificity of our results in distinguishing healthy cognition from AD-induced cognitive decline and dementia progression, and the integration of MMSE and MoCA data allowed us to explore potential causal relationships between lipid and other metabolic dysregulations and AD progression. However, there are some obvious limitations in this study: firstly, the clinical data we have collected are limited, so we cannot explore the relationship among PI, AD, atherosclerosis and hyperlipidemia; in addition, if the observation time could be longer, we could more accurately understand how dyslipidemia changes with time, and if we add resources from the gene database, we could enhance our statistical ability to help us find important genetic connections; finally, if we could test the models of the 16 metabolic indexes in different patient groups in other institutions, the applicability of the models could be wider and the results could be more universal.

## 5. Conclusions

This study profiled plasma metabolomes in 172 Chinese participants across clinical stages of AD. Using untargeted plasma metabolomics in 172 participants, we identified distinct metabolic signatures associated with disease progression. Lipid metabolism, particularly glycerophospholipid and sphingolipid pathways, was profoundly dysregulated. Key metabolites, including ceramides, phosphatidylinositol (PI 38:4), and glucose, correlated significantly with cognitive decline. A 16-metabolite panel effectively distinguished early AD/MCI from controls (AUC = 0.804). Additionally, phosphatidylcholine 42:4 and phosphatidylinositol 34:2 showed positive correlations with MMSE and MoCA scores. These findings support plasma metabolomics as a non-invasive tool for early and differential diagnosis and staging of AD. Longitudinal validation in multi-center cohorts is warranted.

## Figures and Tables

**Table 1:** Demographic and clinical characteristics of study participants.

Basic Information		HC	AD	MCI	SCD	VCI
		N = 33	N = 60	N = 9	N = 23	N = 47
Gender, Male/Female (n/n)		20/13	30/30	7/2	12/11	23/24
Age, Mean (SD)		65.9 (9.0)	66.9 (9.9)	62.2 (9.4)	64.0 (12.3)	69.3 (12.5)
Disease Stage, n	Mild	-	18	-	-	-
	Moderate	-	26	-	-	-
	Severe	-	16	-	-	-

Data are presented as counts or mean ± standard deviation (SD). Between-group comparisons were performed using independent *t*-tests for continuous variables and chi-square or Fisher’s exact tests for categorical variables. Abbreviations: HC, Healthy Control; AD, Alzheimer’s Dementia; MCI, Mild Cognitive Impairment; SCD, Subjective Cognitive Decline; VCI, Vascular Cognitive Impairment and Other Cognitive Disorders.

**Table 2:** Agreement between the 16-metabolite panel and the reference standard.

	AD+MCI (Positive)	HC+VCI+SCD (Negative)	Total
Positive	43	26	69
Negative	20	83	103
Total	63	109	172

Note. Sensitivity = 62.3% (95% CI: 50.75–73.75%); Specificity = 80.6% (95% CI: 72.04–88.07%%). PPV = 68.3%; NPV = 76.1%.

## Data Availability

The raw metabolomic datasets generated and analyzed during this study are not publicly available due to patient privacy and ethical restrictions imposed by the Institutional Review Board of Peking University Shenzhen Hospital (approval number: 2024-151). However, de-identified processed data supporting the findings of this study are available from the corresponding author upon reasonable request, subject to IRB approval and compliance with the Declaration of Helsinki.

## Supplementary Materials

The following supporting information can be downloaded at: https://www.mdpi.com/article/10.3390/metabo16060377/s1, Figure S1: Study flowchart.
