# Supplementary figures and images for: Application of Plasma Metabolomic Biomarker Panels in Early Diagnosis and Disease Staging of Alzheimer’s Disease
Source: Metabolites. 2026 May 30;16(6):377. doi: 10.3390/metabo16060377 (PMC13302915; doi:10.3390/metabo16060377)

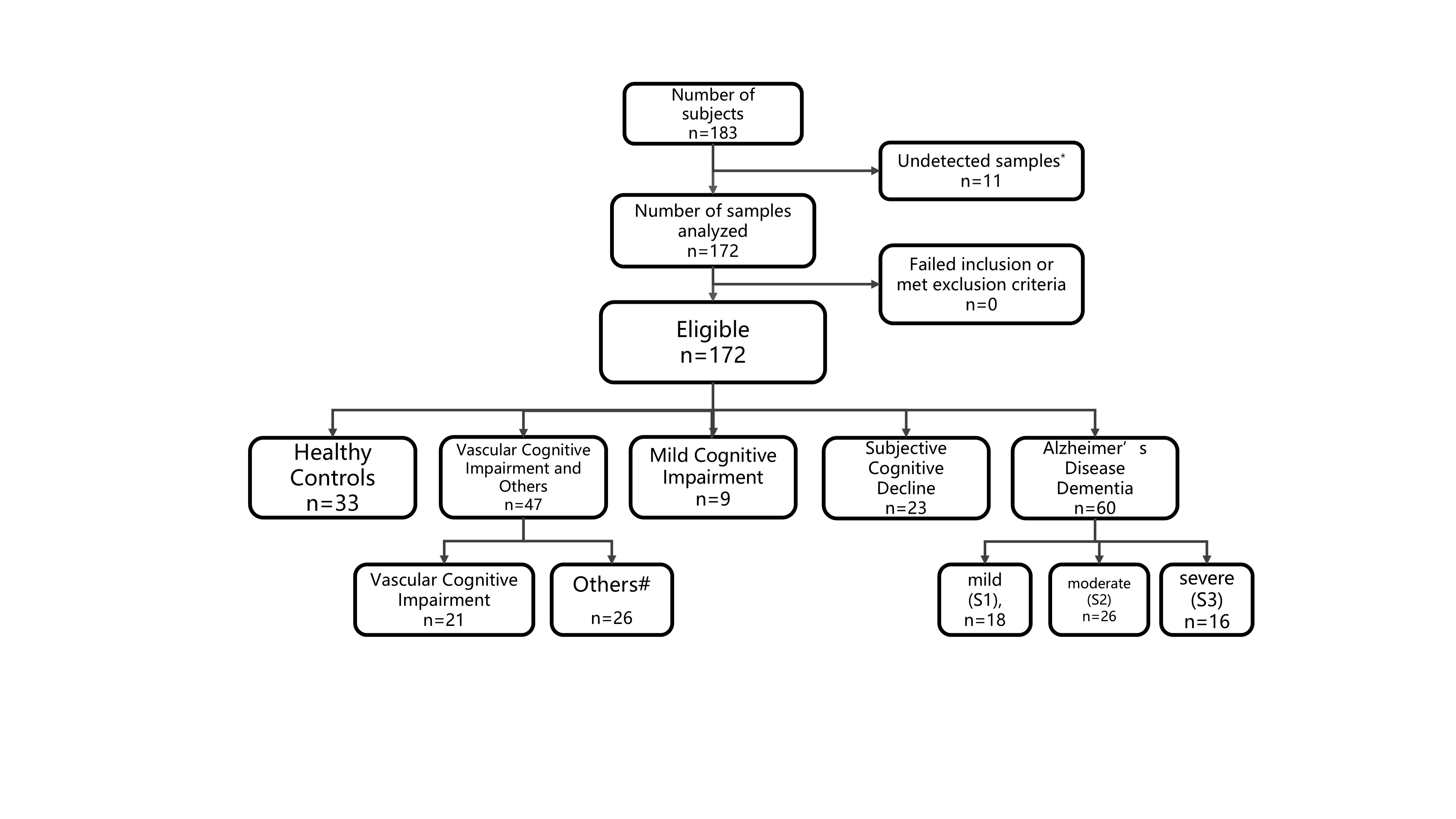

Supplement: Supplementary file 1 [file metabolites-16-00377-s001.zip › metabolites-4308218-supplementary.jpg]
